# Decoding collective communications using information theory tools

**DOI:** 10.1098/rsif.2019.0563

**Published:** 2020-03-18

**Authors:** K. R. Pilkiewicz, B. H. Lemasson, M. A. Rowland, A. Hein, J. Sun, A. Berdahl, M. L. Mayo, J. Moehlis, M. Porfiri, E. Fernández-Juricic, S. Garnier, E. M. Bollt, J. M. Carlson, M. R. Tarampi, K. L. Macuga, L. Rossi, C.-C. Shen

**Affiliations:** 1 Environmental Laboratory, U.S. Army Engineer Research and Development Center (EL-ERDC), Vicksburg, MS, USA; 2 EL-ERDC, Newport, OR, USA; 3 National Oceanic and Atmospheric Administration, Santa Cruz, CA, USA; 4 University of California, Santa Cruz, CA, USA; 5 Department of Mathematics, Clarkson University, Potsdam, NY, USA; 6 School of Aquatic and Fishery Sciences, University of Washington, Seattle, WA, USA; 7 Department of Mechanical Engineering, University of California, Santa Barbara, CA, USA; 8 Department of Mechanical and Aerospace Engineering and Department of Biomedical Engineering, New York University Tandon School of Engineering, Brooklyn, NY, USA; 9 Department of Biological Sciences, Purdue University, West Lafayette, IN, USA; 10 Department of Biological Sciences, New Jersey Institute of Technology, Newark, NJ, USA; 11 Department of Physics, University of California, Santa Barbara, CA, USA; 12 Department of Psychology, University of Hartford, West Hartford, CT, USA; 13 School of Psychological Science, Oregon State University, Corvallis, OR, USA; 14 Department of Mathematical Sciences, University of Delaware, Newark, DE, USA; 15 Department of Computer and Information Sciences, University of Delaware, Newark, DE, USA

**Keywords:** collective behaviour, mutual information, transfer entropy, causation entropy

## Abstract

Organisms have evolved sensory mechanisms to extract pertinent information from their environment, enabling them to assess their situation and act accordingly. For social organisms travelling in groups, like the fish in a school or the birds in a flock, sharing information can further improve their situational awareness and reaction times. Data on the benefits and costs of social coordination, however, have largely allowed our understanding of why collective behaviours have evolved to outpace our mechanistic knowledge of how they arise. Recent studies have begun to correct this imbalance through fine-scale analyses of group movement data. One approach that has received renewed attention is the use of information theoretic (IT) tools like *mutual information*, *transfer entropy* and *causation entropy*, which can help identify causal interactions in the type of complex, dynamical patterns often on display when organisms act collectively. Yet, there is a communications gap between studies focused on the ecological constraints and solutions of collective action with those demonstrating the promise of IT tools in this arena. We attempt to bridge this divide through a series of ecologically motivated examples designed to illustrate the benefits and challenges of using IT tools to extract deeper insights into the interaction patterns governing group-level dynamics. We summarize some of the approaches taken thus far to circumvent existing challenges in this area and we conclude with an optimistic, yet cautionary perspective.

## 1. Introduction

Collective motion is an adaptive strategy found across multiple scales of biological organization, from cellular migrations to crowds of pedestrians [1–4]. Consequently, research on this subject is generally interdisciplinary and provides insights into a broad range of socially mediated actions, such as resource acquisition, navigation, risk mitigation and even basic democratic principles [5]. Yet, while few assumptions are required to model collective motion, the search for general ‘rules’ that govern these dynamical systems continues [6–9].

Ecological and evolutionary factors have played an important role in generating the algorithms governing collective behaviour, but these forces can also be a source of consternation for investigators. Collective actions often rely on social cues, which are inherently ambiguous and ephemeral, and the benefits associated with group membership are context-dependent and can quickly become costs (e.g. improved resource acquisition and vigilance versus density-dependent competition or energetic losses from false alarms) [10]. While the macroscopic patterns of such collective actions are well documented across a wide array of taxa (bacteria [11], insects [12], fish [13], birds [14] and humans [15]), the nature of the inter-individual interactions driving them remains an open question [16,17]. Specifically, while many studies are able to infer interactions among group members (e.g. [6,18–23]) it remains challenging to synthesize these lessons across bodies of work as approaches and conclusions can vary on a case-by-case basis.

Recently, there has been a renewed interest in the application and development of information theoretic (IT) tools to study interaction patterns in both real and synthetic data. IT tools are being used to identify statistical structures, information flow and causal relationships on topics ranging from societal trends [24] to leader–follower dynamics [23,25–28] and predator–prey interactions [29]. IT tools are well suited for characterizing statistical patterns in time varying, dynamical systems and they have played a prominent role in doing so across a range of disciplines [30–32]. However, while there are seminal sources on information theory and the metrics derived from Claude Shannon’s work (e.g. [33,34]), these are often tailored to specific disciplines and can be challenging to interpret and apply due to a failure to recognize either the mathematical or biological conditions involved in a given process [35–38]. Consequently, we find the recent application of IT tools in collective behaviour to be skewed towards the physical and mathematical disciplines. The extent to which these tools can help ecologists, psychologists and evolutionary biologists studying collective behaviour remains unknown.

The goal of this paper is to provide a brief, practical synthesis on the benefits and pitfalls of applying IT tools like *mutual information*, *transfer entropy* and *causation entropy* to quantify interaction patterns in groups on the move. We begin by highlighting the utility of IT tools through a series of examples designed to introduce each of the above metrics in the context of modelling interactions in group movement data. We then use concrete examples to demonstrate the benefits and pitfalls of applying IT tools to this type of data. We conclude by summarizing common challenges in the application of these tools and discuss their future potential for the study of collective behaviour.

## 2. Decoding collective communications

Information is a fitness-enhancing currency, as all organisms share essential needs in terms of acquiring resources, mitigating risk, and reducing uncertainty. The definition of information in animal communications, however, remains a contentious topic in biology and how ‘information’ is defined often clashes with Shannon’s original definition [10,35,39]. Shannon himself had warned his peers against what he saw as the growing misuse of the theory across disciplines (the bandwagon effect) [40]. Nonetheless, IT approaches are rooted in statistical mechanics [41] and therefore have the potential to provide a model-free means of quantifying statistical associations between data streams, such as the positions or orientations recorded between individuals over time.

Before proceeding, it is worth clarifying why one should consider using IT tools if there is a chance of getting misleading information. After all, there is already a rich variety of approaches used to quantify inter-individual interactions (e.g. pair-wise correlations [18,19], force-matching [20], mixed-models [42], neural networks [43] and extreme-event synchronization [44]). This methodological diversity holds the promise of providing robust insights when different approaches converge on similar conclusions.

There is also merit in adopting common quantities that can improve current efforts to make comparisons across studies or systems in the absence of a consensus. Consider the order parameter. It is an intuitive quantity that characterizes a group’s coordination (or order) and has been widely adopted, yet it tells us nothing about the underlying interactions that have generated the observed behaviours. IT tools have the potential to fill this gap in a number of ways. Mutual information provides an equitable means of drawing statistical comparisons between variables that correlations can misconstrue [45–48]. Transfer entropy enables one to isolate influential interactions [49] and causation entropy can distinguish between direct and indirect influence [50,51]. Below we demonstrate each of these properties and we begin by reviewing the source of these metrics, Shannon’s entropy.

### 2.1. Information and Shannon entropy

The foundation of modern information theory is Shannon’s notion of entropy [33], which measures a stochastically fluctuating observable and was originally used to quantify the uncertainty found in decoding a message. The heading of a bird in a flock, for example, is a measurable quantity that will fluctuate due to the complex moment-to-moment decisions a bird must make in response to the movements of its neighbours; the variability of this quantity can thus be characterized within Shannon’s formalism. More generally, for a discrete random variable *X* with domain *x* ∈ *X* and probability mass function *p*(*x*), Shannon defined the information contained in outcome *x* as − log *p*(*x*). He selected this logarithmic characterization so that the information of statistically independent outcomes would be additive. The expected value of this quantity over the distribution *p*(*x*) is called the Shannon entropy, *H*(*X*), so named because, similar to its thermodynamic counterpart, it can be interpreted as an average measure of the amount of uncertainty or ‘disorder’ in variable *X*

2.1 $$H(X)\equiv -\sum_{x\in X}p(x)\log p(x).$$

By convention, one sets 0 log 0 equal to zero so that impossible outcomes do not cause the sum to diverge. The Shannon entropy is formally a dimensionless quantity, but, because the base of the logarithm is arbitrary, it is customary to refer to different choices of this base as different ‘units’. For instance, if the natural logarithm (base *e*) is used, the entropy is said to be in units of nats, but if the base two logarithm is used, the units are in bits.

As a concrete example, consider the Shannon entropy of a coin toss that comes up heads with probability *p*. Applying equation (2.1), one finds that

**Figure 1.**
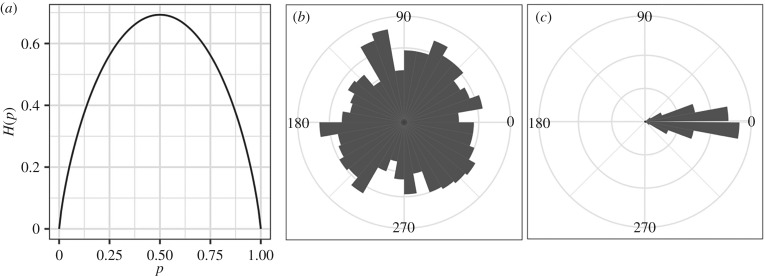
Shannon entropy. (*a*) The Shannon entropy in nats for the simplest case of only two possible outcomes, *p* and 1 − *p* [33]. (*b*,*c*) Illustration of how the Shannon entropy of a standard movement variable, individual orientation, can vary with ecological context. A group of animals can show greater variability in their orientations as they forage semi-independently of one another in an area (*b*), but this variability can drop as they travel together towards a common goal (*c*). On average, the change in variability between (*b*) and (*c*) corresponds to a drop in Shannon entropy, in nats, from (*b*) *H*(*X*) = 2.04 ± 0.006 to (*c*) *H*(*X*) = 1.32 ± 0.03. Estimates of *p*(*x*) were calculated from 1000 random samples of *X*, drawn either from a uniform distribution bounded by [0, 2*π*] or a Gaussian one (mean = 0, sd = 0.25) modulo 2*π*. Values of *x* were then sine transformed and binned to estimate *p*(*x*). Bin widths were defined by the optimal width determined in the uniform distribution using the Freedman–Diaconis algorithm [52]. We then replicated this process 1000 times to estimate the mean and standard deviation of *H*(*X*) for each distribution.

2.2 H(p)=−plog⁡p−(1−p)log⁡(1−p),

which we plot in figure 1*a*. The Shannon entropy is maximized when the coin toss is fair (*p* = 1/2); it is minimized when the coin is weighted to always come up one way or the other (*p* = 0 or *p* = 1). In general, for a given possibility space, the Shannon entropy is maximal when all outcomes are equally likely and decreases as probability is increasingly biased towards a particular outcome or subset of outcomes. The link between the uniformity of a probability distribution and our intuition for entropic disorder is further illustrated in figure 1 in the case of context-dependent movement patterns [53]. Consider a group of social animals meandering around as they forage within a resource patch (figure 1*b*) versus when they travel together between destinations (figure 1*c*). In the former condition, individuals are more prone to stochastic movements as they search for food, meaning that the probability of finding an individual with any instantaneous heading is roughly uniform. During the latter, group members share a common directional goal as they travel together, so this probability becomes heavily concentrated around the direction of collective motion. In this context, we can show that condition 1 (*b*) displays a greater amount of Shannon entropy than condition 1 (*c*), as it is harder to guess any given animal’s direction from one instant to the next in the former case than in the latter.

### 2.2. Mutual information

Because the study of collective motion is broadly concerned with connecting group behaviour to the local interactions of group members, we are often less interested in the absolute uncertainty in some observable quantity than we are in the extent of its correlation with another. If two observables *X* and *Y* are statistically independent, then their joint distribution *p*(*x*, *y*) will just equal *p*(*x*) *p*(*y*), allowing us to generalize equation (2.1) to define a joint entropy *H*(*X*, *Y*) = *H*(*X*) + *H*(*Y*). If there is some statistical correlation between these observables, however, then *H*(*X*, *Y*) will be reduced by some amount MI(*X*; *Y*):

2.3 MI(X;Y)≡H(X)+H(Y)−H(X, Y).

We refer to this quantity as the mutual information between variables *X* and *Y*, and it is a measure of how much our uncertainty in one variable is reduced by knowing the other. When *Y* is known, the probability of *X* is given by the *conditional* distribution *p*(*x*|*y*), so the mutual information can also be expressed as the difference between *H*(*X*), the total uncertainty in *X*, and the conditional entropy *H*(*X*|*Y*). The reduced uncertainty in *X* when *Y* is known is

2.4 MI(X;Y)=H(X)−H(X|Y)=H(Y)−H(Y|X).

The second equality above follows from the fact that equation (2.3) is unchanged if *X* and *Y* are swapped, a symmetry that exists because MI measures correlation and not causation.

Measuring correlations between variables is one of the most commonly employed, and often insightful, tools used in the study of collective motion (e.g. [6,7,18,19]). Yet, individuals travelling in groups have been shown to display high-dimensional interactions [43], which increases the chances of uncovering nonlinear associations between observables. Dynamic interactive systems are often replete with nonlinear correlations that typical comparative metrics, like the covariance or Pearson’s correlation coefficient, can misconstrue because these quantify linear relationships. Sometimes these comparative metrics can help uncover the underlying relationship between two observables, but this is not always the case.

**Figure 2.**
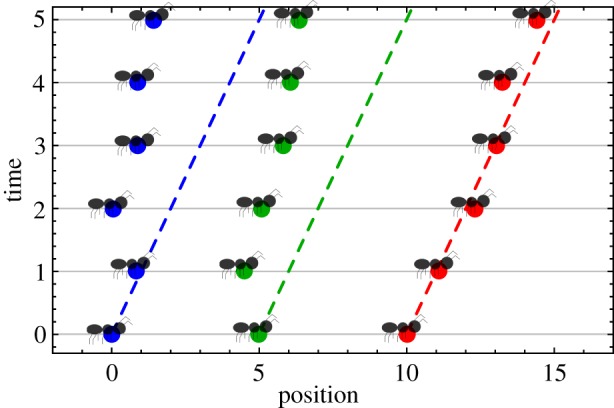
Ant model trajectory. A sample trajectory of the ant model used as an example throughout this section. The position of each of the three ants is given on the abscissa, and each horizontal slice shows the configuration of the system at different times (both measured in arbitrary model units). Each dashed line shows what the trajectory of the like-coloured ant would be in the absence of any stochasticity. Note how the middle ant (green) initially takes a step backwards, which results in its follower (blue) making a backwards move one time step later.

The way in which mutual information quantifies correlation is fundamentally different from that of other common comparative metrics, such as the covariance, and we can illustrate this with a simple example. Consider a scenario in which we have data on the movements of a group of ants out on a foraging expedition from their colony. We would like to better understand how the inter-individual interactions contribute to the group’s movement patterns. We can model this as a one-dimensional process in which we define the position of the lead ant at time *t* to be *x*_0_(*t*) and the position of each subsequent ant in line as *x*_1_(*t*), *x*_2_(*t*), etc. We can begin simply by modelling the lead ant as marching according to a Gaussian random walk with mean step size Δ*X* and standard deviation *σ*. If our ants are related to one another and thus share the same behavioural circuitry, we may expect each ant to more-or-less match the kinetic actions of its predecessor. As such, we will assume that each ant attempts to choose its next step to match the size of the most recent step taken by the ant preceding it. Owing to sensory or mechanical error, this will be a stochastic process, and, for simplicity, we will assume that it can also be modelled as a Gaussian random walk with the same variance as the walk performed by the lead ant. The mean step size for ant *n* ≠ 0 at time *t*, however, will be equal to the size of the actual step taken by the ant in front of it a moment earlier (*n* − 1 at time *t* − 1; see figure 2 for an illustration of the model).

One of the simplest comparisons we can make in this example is between the position of the *n*th ant before and after taking a step, i.e. compare the random variables *X*_*n*_(*t*) and *X*_*n*_(*t* − 1). In our example, the easiest way of quantifying the correlation between these variables is the covariance

**Figure 3.**
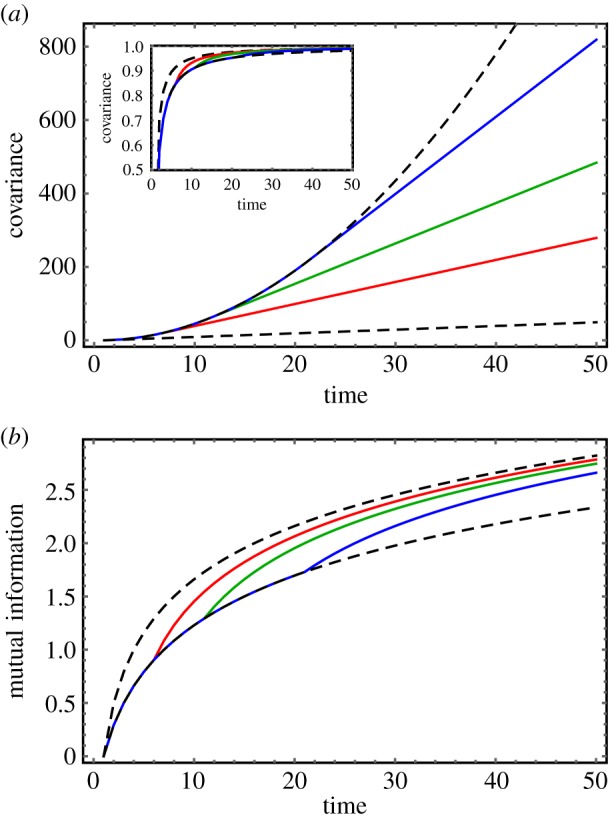
Covariance versus mutual information. (*a*) cov(*X*_*n*_(*t*), *X*_*n*_(*t* − 1)) for the ant model with *n* = 5 (red), 10 (green) and 20 (blue). The bottom dashed curve is the covariance for *n* = 0 (the lead ant), which serves as a lower bound. The upper dashed curve is the common curve that each covariance obeys for 0 < *t* ≤ *n*. (*b*) MI(*X*_*n*_(*t*); *X*_*n*_(*t* − 1)) in units of bits for the same set of values of *n*. The order of the curves here is reversed, with the *n* = 0 curve now serving as an upper bound. The inset of (*a*) shows that the same ordering can be achieved with the covariance if it is normalized by the product of the standard deviations in *X*_*n*_(*t*) and *X*_*n*_(*t* − 1), but at the cost of compressing the curves to the unit interval.

2.5 $$\mathrm{cov}(X_{n}(t),X_{n}(t-1))=\langle \delta X_{n}(t)\delta X_{n}(t-1)\rangle ,$$

where 〈*X*〉 is the expectation value of the random variable *X*, and *δX* ≡ *X* − 〈*X*〉. With a few reasonable assumptions, both the covariance and the mutual information of the random variables *X*_*n*_(*t*) and *X*_*n*_(*t* − 1) can be evaluated analytically for this model, and we compare these metrics in figure 3 as functions of time, for several different values of *n*.

Intuitively, we anticipate that the uncertainty in the size of each step an ant takes should be limited, whereas the uncertainty in the ant’s absolute position (the summation of a growing number of stochastic steps) should increase steadily with time. This implies that an ant’s current and previous position, which are linked by a single step, must become increasingly correlated over time, which the covariance and mutual information both confirm. They also both agree that the rate of this growth will be the same for all ants until time *t* = *n*, which is the time it takes for information (i.e. uncertainty) about the lead ant’s step size to propagate down the file to the *n*th ant.

Where the two metrics differ most starkly, however, is in their characterization of how correlation varies across the chain at long times. The covariance indicates that for sufficiently long times, it will grow as one moves towards the back of the line, and the rate of this growth increases without bound as time passes. The mutual information, on the other hand, decreases down the chain, after long enough times, and the rate of this decrease gets progressively smaller with time. The origin of this discrepancy is an apple to oranges comparison. This example highlights that both metrics quantify correlation, but they are measuring correlations between a different pair of variables. The covariance cov(*X*, *Y*) estimates the correlation between the variables *X* and *Y*, whereas the mutual information MI(*X*; *Y*) actually compares the variables *X* and *X*|*Y* (*X* conditioned on *Y*).

In our toy movement model, although both the current and previous positions of ant *n* become increasingly uncertain as *n* increases down the chain, they always remain, on average, within a distance Δ*X* of one another. This results in a growing correlation between the variables *X*_*n*_(*t*) and *X*_*n*_(*t* − 1) as one moves down the chain, consistent with the trend observed for the covariance. The mutual information, however, accounts for the fact that as *n* increases, growing uncertainty in the step size limits the extent to which knowing *X*_*n*_(*t* − 1) can reduce our uncertainty in *X*_*n*_(*t*). Thus the mutual information must ultimately decrease as one goes down the chain. (If it did not, this system would violate the data-processing inequality [34].) Put another way, whereas the covariance measures correlations between the current and previous positions of ant *n*, the mutual information measures correlations between the position of ant *n* and the size of its steps.

As we mentioned earlier, comparative metrics like Pearson’s correlation can effectively capture linear associations. In our simple example, the covariance can also be made to decrease down the chain if it is normalized by the product of the standard deviations in *X*_*n*_(*t*) and *X*_*n*_(*t* − 1) (see the inset of figure 3*a*), but this results in a different apples to oranges comparison by mapping the values of the covariance from the open interval [0, ∞) (same as the MI) to the closed interval [0, 1]. Recall that more complex systems often exhibit higher order, nonlinear correlations that cannot be captured by the covariance, whether it is normalized or not. The mutual information, however, has been proven to capture all higher order correlations between two random variables [45–47].

Like other measures of correlation, mutual information is principally a comparative metric, best suited for probing the *relative* strength of statistical associations. While one bit of information can be concretely characterized as the amount needed to encode a single, unweighted binary decision (yes/no, on/off), mutual information cannot determine *which* bits of information two variables share. Nonetheless, MI has proven to be rather useful in a range of relevant settings, from identifying critical phase transitions in a flocking model [54], measuring the complexity of visual cortical networks [55] and linking neural firing rates with motor actions [56,57].

### 2.3. Transfer entropy

The symmetric structure of mutual information (equation (2.3)) precludes it from measuring directed influence. Two birds both following the same leader will have highly correlated motion, but they may not directly influence one another. To determine whether one random variable is actually influenced by another, rather than merely correlated with it, one can instead use an IT metric called transfer entropy (TE) [49]. This metric has been used to measure how movement information cascades through simulated [25,58] and real [27,59] systems, to study interactions between animals and robots [29,60,61], and to establish hierarchy in leader–follower experiments [23,44].

Given two random variables *X*(*t*) and *Y*(*t*) whose values change stochastically with time (we call such a variable a *stochastic process*), we define the transfer entropy from *Y* to *X* in terms of Shannon entropies as follows:

2.6 $$\begin{array}{rl} {\text{TE}}_{Y\to X} & =H(X(t)|{\left\{X(\tau )\right\}}_{\tau })-H(X(t)|{\left\{X(\tau )\right\}}_{\tau },{\left\{Y(\tau )\right\}}_{\tau })\\ & =\text{MI}(X(t)|{\left\{X(\tau )\right\}}_{\tau };{\left\{Y(\tau )\right\}}_{\tau }), \end{array}$$

where {*X*(*τ*)}_*τ*_ is the past trajectory of *X*, i.e. the set of all values of *X*(*τ*) for all *τ* < *t*. As the second equality shows, transfer entropy is just the mutual information shared between the process *X* at time *t*, conditioned on its past, and the past trajectory of the process *Y*(*t*). Equation (2.6) seems rather complicated to evaluate, but in most applications time will be discretized and the processes involved will be well approximated as Markovian, meaning that the set {*X*(*τ*)}_*τ*_ will only include the value of *X* at its previous time step. Arguably, this assumption is often made more for mathematical simplicity and tractability than for biological realism and we return to the topic of defining *τ* later when discussing sampling intervals in §3.3. Even when the Markov property does not hold, it may seldom be necessary to condition over every previous time point. For example, in the ant model introduced in the previous subsection, the transfer entropy from *X*_*n*−1_(*t*) to *X*_*n*_(*t*) only requires conditioning on the previous value of *X*_*n*_ and the previous two values of *X*_*n*−1_, since the *n*th ant chooses its step size (*x*_*n*_(*t*) − *x*_*n*_(*t* − 1)) based on the previous step size of the ant in front of it (*x*_*n*−1_(*t* − 1) − *x*_*n*−1_(*t* − 2)).

**Figure 4.**
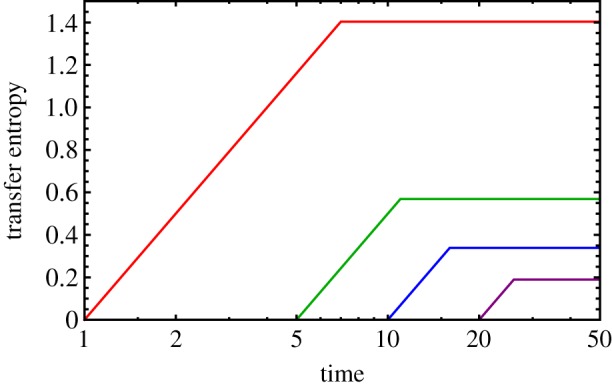
Transfer entropy between different pairs of ants. The transfer entropy ${\text{TE}}_{X_{5}\to X_{n}}(t)$ is plotted in units of bits for the ant model for *n* = 6 (red), 10 (green), 15 (blue) and 25 (purple). The transfer entropy in each case is zero for 0 < *t* ≤ *n* − 5 and then increases logarithmically until saturation occurs for *t* > *n*. (Note that the abscissa is on a log scale to make the logarithmic growth look linear.)

If two processes *X*(*t*) and *Y*(*t*) are merely correlated, the past trajectory of *Y* will tell us nothing more about *X*(*t*) when the past trajectory of *X* is already known. It is only when *Y* directly influences the dynamics of *X* that there will be a nonzero transfer entropy TE_*Y*→*X*_. This kind of influence is often interpreted as a flow of information from process *Y* to process *X*, and our ant model provides an excellent visualization of this flow. Figure 4 plots the transfer entropy from ant *m* to ant *n* for *m* = 5 and several different values of *n* > *m*. It takes *n* − *m* time steps for any information to flow from ant *m* to ant *n*, so the transfer entropy is zero until then. After that, the transfer entropy begins to increase as information from further forward in the chain passes through ant *m* and arrives at ant *n*. After *n* time steps, information from the lead ant finally arrives, and the transfer entropy levels off because there is no further information to transmit. Note that because transfer entropy measures *directed* influence, it is an asymmetric quantity, and the transfer entropy flowing from site *n* back towards site *m* is always zero.

### 2.4. Causation entropy

If transfer entropy distinguishes influence from correlation, it would be useful to have a metric that further distinguishes direct and indirect influence. In our ant model, for example, each ant is indirectly influenced by every ant ahead of it, but it is only directly influenced by the ant in front of it. We can determine whether ant *m* directly influences ant *n* by asking the following question: if the past trajectory of every ant besides ant *m* is already known, is there anything more that the past trajectory of ant *m* can tell us about the position of ant *n*? If so, then ant *m* has a unique and therefore direct influence on the motion of ant *n*. Mathematically, the answer to this question can be quantified as a generalization of the transfer entropy known as causation entropy (CSE) [50,51]

2.7 $${\text{CSE}}_{X_{m}\to X_{n}}=H(X_{n}(t)|{\left\{X_{\;j}(\tau )\right\}}_{\;j\neq m,\tau })-H(X_{n}(t)|{\left\{X_{\;j}(\tau )\right\}}_{\;j,\tau }).$$

**Figure 5.**
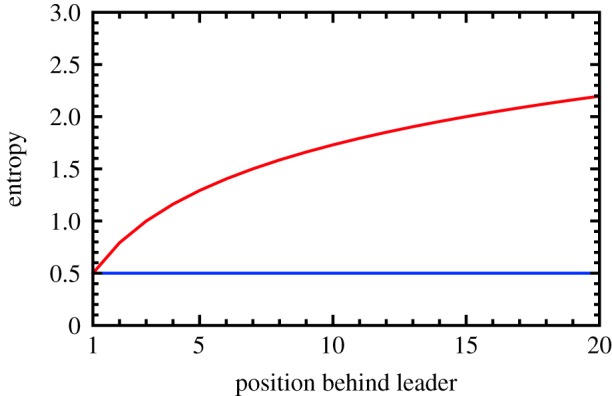
Comparison of transfer and causation entropy. The long-time limiting value of the transfer entropy ${\text{TE}}_{X_{n-1}\to X_{n}}$ and the *t* > 1 value of the causation entropy ${\text{CSE}}_{X_{n-1}\to X_{n}}$ are plotted in red and blue, respectively, for the ant model as a function of the position behind the leader (*n* > 0) in units of bits. The constant CSE for each pair of adjacent ants is a reflection of the homogeneity of the model’s rule-based interactions. Any differences in the dynamics of each ant derive from the indirect influence of the ants further up front, which is reflected in the growth of the TE curve as that number increases. The logarithmic character of this growth suggests that the efficiency of indirect influence falls off with distance.

In the above, {*X*_*j*_(*τ*)}_*j*≠*m*,*τ*_ is to be understood as the past trajectories of all ant positions except *X*_*m*_. It is straightforward to demonstrate that the causation entropy in equation (2.7) is equal to 1/2 bit for *m* = *n* − 1, *t* > 1 and is zero otherwise. This result is sensible, since only neighbouring ants directly interact, and the interactions were assumed to be uniform across the chain. It takes exactly one time step for information to flow from an ant to the one directly following it, hence why there is only causation entropy for *t* > 1. Long time differences in the step-size variations of each ant are due entirely to the indirect influence of ants further ahead in line, as illustrated in figure 5.

Application of CSE to group movement data has clear potential in terms of testing proposed theories for the various ‘rules’ governing how group members coordinate their movements. For instance, Lord *et al.* [26] found that midges in a swarm did not solely rely on their nearest neighbours for navigational guidance. Rather, midges appear to have causal connections that extended further than would be expected from a strict, or even lax, nearest neighbour rule.

**Figure 6.**
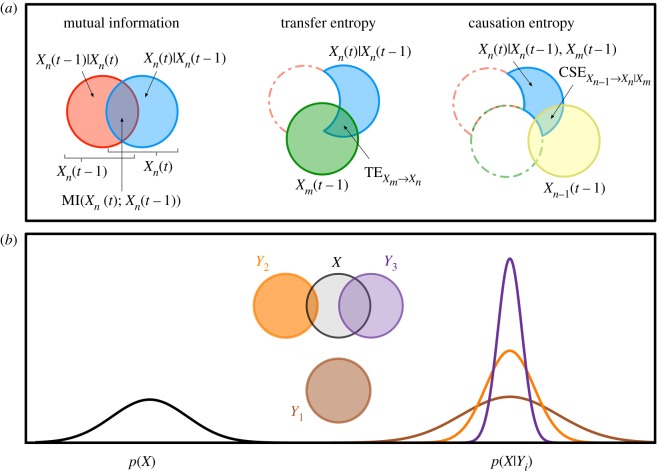
Summary of IT metrics. (*a*) The three IT metrics calculated for the ant model are summarized here in standard Venn diagram format. Note how in going from mutual information to causation entropy, the uncertainty in the variable *X*_*n*_(*t*) is successively reduced by conditioning it on more and more variables. (*b*) All the IT metrics presented in this section make comparisons between a distribution of one variable, generically *p*(*X*), and a second distribution of that variable conditioned on another, *p*(*X*|*Y*_*i*_). The size of the corresponding IT metric depends upon how much that conditioning reduces the uncertainty in *X*, which, for the normally distributed random variables in the ant model, is directly related to how much conditioning reduces the width (variance) of the distribution. In the Venn diagram, this reduction corresponds with the extent to which the area of the circle representing *X* is reduced by its overlap with the circle representing *Y*_*i*_.

An illustration of the IT metrics of mutual information, transfer entropy and causation entropy may be found in figure 6*a* using standard Venn diagrams. In figure 6*b*, we illustrate how the degree of overlap between circles in a Venn diagram relates to the reduction in the uncertainty of a given random variable.

## 3. Identifying communication patterns

Armed with a short primer on how IT metrics like *mutual information*, *transfer entropy* and *causation entropy* can be useful in the analysis of movement data, we now illustrate how our previous examples relate to real-world challenges in the study of collective motion. In particular, we wish to contrast two examples designed to highlight the potential benefits and pitfalls of applying IT tools to group movement data. We begin by illustrating a condition in which these tools can be used in concert to provide a deeper understanding of the inter-individual interactions governing observed group-level dynamics. We then use the example of studying social recruitment to demonstrate how IT tools are sensitive to sample size. To help place these examples into a broader context, we preface each with their ecological relevance and touch on some current approaches.

### 3.1. Inferring local interactions from global patterns

**Figure 7.**
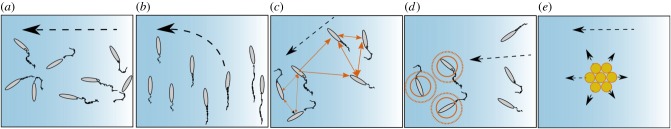
Collective gradient climbing strategies. (*a*) When attempting to climb an environmental gradient (intensifying from white to blue) individual errors can be cancelled out, and, by staying together, a group’s mean direction (dashed arrow) can lead up the gradient (the many-wrongs hypothesis) [66,67]. (*b*) When individuals in preferred locations slow down relative to those in lower quality regions (shown by shorter trailing wakes), social cohesion can combine with the resultant speed differential to rotate the group up the gradient and lead to a case of emergent sensing [68]. (*c*) In theory, we may also expect variable interaction strengths based on local conditions (line/arrow thickness). Individuals further up the gradient are less social as they take advantage of their location in the gradient, while individuals in less favourable locations show stronger social ties as they seek better locations along the gradient [69]. (*d*) If individuals are cooperating, those located up a gradient could also signal to others the quality of the resource based on a local scalar measure (circles) [70]. (*e*) The preceding examples may also apply at the cellular level. Cell clusters are polarized radially, with each cell producing a force in the direction of polarization, resulting in a net motion up the gradient similar to (*a*) [71].

Understanding why and how animal groups move the way they do often begins by looking for patterns in observable quantities, like the positions or orientations of individuals, and quantifying how these change across a group with time or ecological context. Comparing how metrics derived from such data, like group density or polarity, differ across conditions can reveal evolutionary adaptive advantages that clarify why such behaviour persists. For instance, social animals often close ranks when threatened, which can reduce the average member’s predation risk (the selfish herd) [62,63] and, when attacked, members may temporarily scatter to avoid capture, thereby further reducing their risk by confusing the attacker (the confusion effect) [64,65]. Inspecting group movement data (either real or synthetic) at a finer spatio-temporal scale has also generated new insights into some of the alternative communication strategies that may govern the movement decisions of group members, as seen in the empirical and theoretical work on how groups may climb environmental gradients (figure 7) [72].

Inferring how individuals are coordinating their movements from group movement data, however, is often problematic since the same group behaviour can be produced by different social interaction strategies or vary with context. For instance, figure 7 illustrates different mechanisms that may all result in the same overall group-level behaviour. A comparable analogy can be made regarding the interaction patterns in flocks of European starlings (*Sturnus vulgaris*). These animals appear to display interactions that are invariant to separation distance while in flight [6,73], but when starlings are foraging rather than flying, their interactions appear to decay non-linearly with increasing separation distance [74]—a pattern that translates more generally across sensory systems [75]. Similar sensitivities can be observed in social fish, where certain variables seem important in driving social interactions under some conditions (e.g. the role of motion cues) [76,77], but not in others [21]. The point is that evidence that is both compelling, yet conflicting, is not uncommon in the study of collective behaviour.

While we do not suggest that IT tools can provide a panacea to explain context-dependencies, their application can certainly be instructive. As an example, we study the dynamics of Tamás Vicsek’s minimal flocking model [78], in which similar group morphologies can emerge from both global and local interaction paradigms, and we demonstrate how IT tools can be used to provide some insights into the nature of the underlying interactions in each case.

Briefly, the Vicsek model consists of *N* agents confined within an *L* × *L* square by periodic boundary conditions. The agents are represented as points whose positions, **r**_*i*_, and orientations, *θ*_*i*_, are randomly assigned at the start of each simulation from uniform distributions [0, L] × [0, L] and [−*π*, *π*], respectively. These particles then move discretely within the domain at a constant speed *v*_*o*_ according to

3.1 $$r_{i}(t+\Delta t)=r_{i}(t)+v_{i}(t)\Delta t,$$

where the particle velocity is **v**_*i*_ = *v*_*o*_ (cos*θ*_*i*_, sin*θ*_*i*_). Particles update their direction of motion by aligning themselves to the (self-inclusive) average orientation of all agents falling within their sensory range, *R*:

3.2 $$\theta_{i}(t+1)=\langle \theta_{i}(t)\rangle_{R}+\Delta \theta_{i}.$$

The first term on the right in equation (3.2) represents the average direction desired and the second is an external noise term that disrupts the accuracy of the process. The value of the noise term is drawn from a uniform distribution [−*ηπ*/2, *ηπ*/2], where 0 ≤ *η* ≤ 2. In this example, we vary only the interaction range parameter, *R*, to contrast two extreme conditions: local versus global interactions. While simplistic, this approach could be extended to contrast observed behaviours against those generated by a null hypothesis.

**Figure 8.**
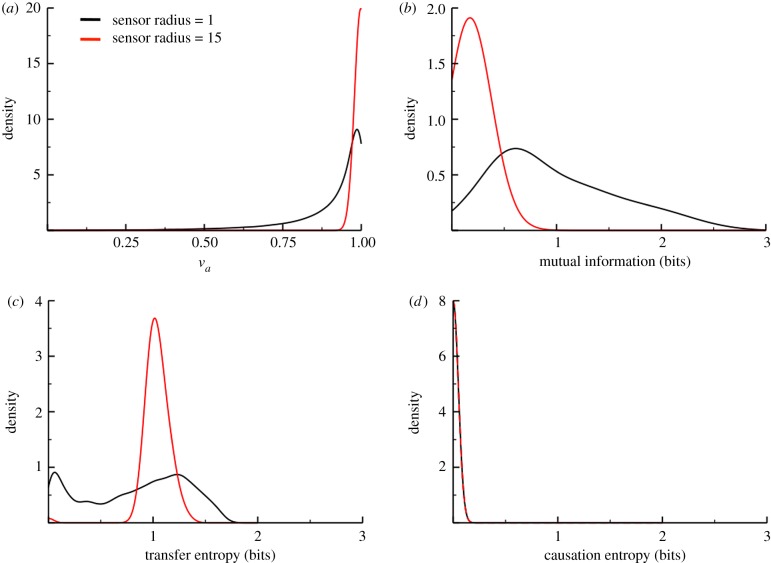
Probability distributions for each sensory scenario. Panels show the distribution of values for the order parameter (*a*), the mutual information (*b*), the transfer entropy (*c*) and the causation entropy (*d*). Curves are coloured by scenario (local interactions, black; global interactions, red). Data were collected from 100 replicate simulations, each of which was allowed to first reach a steady state. For each replicate, IT metrics were computed for each pair of agents, and the results were compiled into the plotted distributions. The Freedman–Diaconis rule [52] was used to histogram all the datasets. Simulation parameters: *N* = 50, *L* = 10, Δ*t* = 1,*v*_*o*_ = 0.1, *R* = 1, 15 and *η* = 10^−3/2^.

We start by using an order parameter, *v*_*a*_, as a comparative measure of the resulting group coordination. It is defined as the magnitude of the summed velocity vectors of all particles, normalized by their number (*N*) and speed (*v*_*o*_), so that its value falls between zero (completely disordered) and unity (perfectly aligned) [78]. We begin by inspecting the distribution of *v*_*a*_ for each condition by computing it for each steady-state time point considered across a hundred replicate simulations and compiling the results (figure 8*a*). Under the global condition, agents are always mutually interacting, so self-assembly is immediate and effectively complete, deviating only slightly due to the small amount of noise added. This results in a distribution that is sharply peaked near *v*_*a*_ = 1. For the local condition, self-assembly occurs through a gradual coarsening of locally aligned clusters, which will not always achieve the same extent of coordination observed in the global condition. This results in a broader distribution.

The order parameter cannot explain *why* one system is less ordered than another, but the mutual information shared between the orientations of any two agents can tell us quite a bit about the underlying dynamics. In figure 8*b*, the narrowly peaked MI distribution of the more ordered condition tells us that knowing the orientation of one agent reduces our uncertainty in that of every other agent by roughly the same extent (the nonzero peak width is due to noise). This is only possible in the case of a spatially uniform, global social interaction. In the less ordered condition, the much broader MI distribution is indicative of interactions that are inhomogeneous. The mean of the distribution is also larger for this condition, which can only be reconciled with its lower orientational order by assuming that a minority of the agents have closely coupled motion, while the majority remain very weakly coupled. This is of course precisely what we know to be true—with local interactions, most agent pairs do not directly interact, which means those that do have a stronger influence over each other’s direction of motion.

We can glean additional dynamical information by studying the corresponding distributions in transfer entropy, which in this context measures the influence that one agent’s current orientation has on the way another will turn. The narrowly peaked TE distribution (the red curve in figure 8*c*) indicates that interactions in the global condition must be symmetrical, precluding any sort of leader–follower type dynamics. The fact that the TE peak is shifted to the right relative to the corresponding MI distribution peak in figure 8*b* also suggests that the uncertainty in each agent’s orientation is reduced more by knowing the prior orientations of other agents than by knowing their current orientations—a consequence of having causal interactions. In the local condition, the black curve in figure 8*c* is roughly bimodal, consistent with our prediction that there should be a mixture of direct and indirect influencers.

Finally, we measure the causation entropy between the orientation of one agent and that of another, conditioned on the orientations of all other agents, to determine how much of a *unique* influence one agent has on another. Figure 8*d* shows that in both systems the distribution of CSE is narrowly peaked at zero. This is unsurprising for the global condition, given what we have already deduced about the uniformity of the interactions; but even with the strongly heterogeneous interactions of the local condition, no agent is uniquely beholden to any other for its decision-making. This begs a couple of interesting questions, though: how many neighbours should an agent pay attention to in order to minimize the amount of redundant information, and how does the agent’s sensory range affect this number?

**Figure 9.**
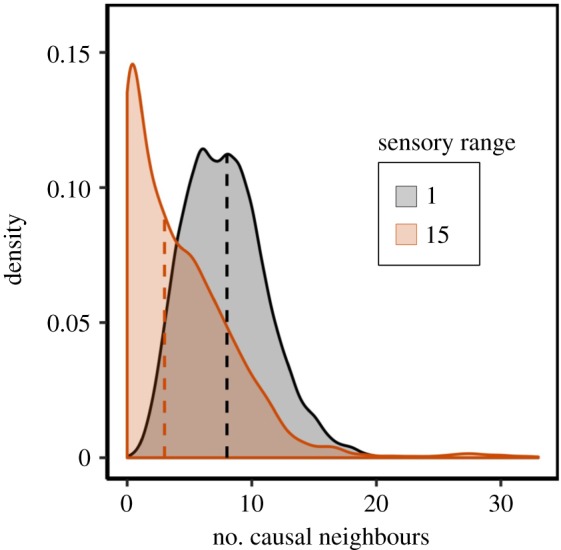
Distribution of causal neighbours. Influential neighbours were identified using the oCSE algorithm (see electronic supplementary material, S1). Dashed lines represent the median value of each distribution (4 and 8 for the global and local scenarios, respectively).

We can answer these questions using a procedure known as the optimal causation entropy algorithm (oCSE), which can find the minimal set of agents whose orientations, if known, maximally reduce the uncertainty in the orientation of some specified agent (for more details, see the electronic supplementary material, S1) [26,51]. Performing this analysis on each of our simulation replicates and compiling the results, we plot the distribution in this optimal number of influential neighbours in figure 9. It seems a bit counterintuitive at first that an agent has notably fewer influential neighbours in the global condition, despite having more neighbours overall; but it is actually consistent with our prior analysis. In particular, the narrowly peaked distribution of the order parameter (figure 8*a*) for the global condition implies that all the agents have orientations that are narrowly distributed about a single global average, meaning that knowing only a few of those orientations will be sufficient to maximally reduce the uncertainty in any other. In the local condition, where the distribution of orientations is broader, more influential neighbours are required to achieve the same reduction in uncertainty.

### 3.2. Tracking information flow

Another strategy to study communication patterns in groups is to look for evidence of social recruitment. That is, identifying whether or not the actions of some individuals provide information that appears to recruit others to do the same (positive feedback). In the case of a group coming to a consensus, the rate at which individuals act (or make a choice) can take on a sharply nonlinear form once a threshold number of group members are involved, which can be considered evidence of a quorum mechanism [79]. In general, evidence for quorum-like processes is widespread and models of varying sophistication that share a sigmoid process have often proven to be a valuable means of characterizing social recruitment (e.g. ants [7,8,80]; cockroaches [81]; fish [8,76,77]; and humans, [3]).

In some cases, the sensory mechanisms driving social recruitment are well established, e.g. quorum sensing in bioluminescent bacteria [82], the chemical signals used by foraging ants [80], or the waggle dance of honeybees [83]. Yet, the flexibility afforded by sigmoid-like functions also underscores the need for caution when interpreting them if the underlying mechanisms are unknown. For instance, recruitment can arise from a simple threshold mechanism rather than the need for a quorum [84] or it may arise strictly due to physical constraints (e.g. density effects from crowding) [4]. In some cases, organisms will simply react differently under different circumstances. Consider that social recruitment in the ant *Temnothorax rugatulus* can change significantly depending on whether the ants are located somewhere familiar (outside their nest) or novel (scouting new locations), even though the same chemical communication signal is employed [85].

If we find evidence of social recruitment in a given scenario, then IT tools would seem to provide a practical means of digging deeper into the underlying interaction patterns governing a recruitment event. However, IT metrics are not infallible, and the estimation accuracy of the values calculated from a dataset can vary considerably. To illustrate this discrepancy, let us consider our ant model from §2, in which we know that each ant recruits another to follow it. In this simple model, we can compute the IT metrics exactly. We can, therefore, compare these expectations with those computed from a statistical analysis of our simulated trajectories.

**Figure 10.**
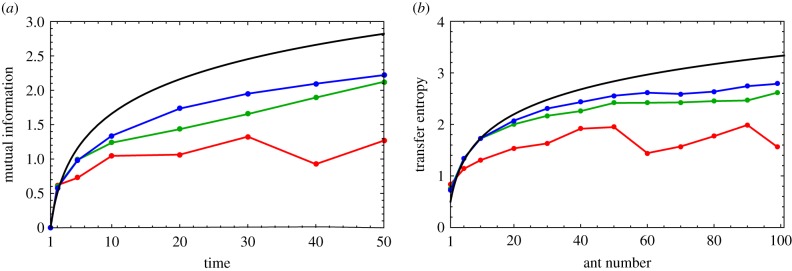
IT metrics: theory versus simulation. (*a*) The mutual information MI(*X*_*n*_(*t*); *X*_*n*_(*t* − 1)) plotted as a function of time. This quantity is independent of *n* for *t* < *n*, allowing for data points for multiple agents at the same time to be compiled. The black curve is the analytic result, and the red, green and blue curves correspond to datasets of 50, 450 and 950 data points, respectively. (*b*) The long-time transfer entropy ${\text{TE}}_{X_{n-1}\to X_{n}}$ plotted as a function of the ant number *n*. The black curve is again the analytic result, and the coloured curves represent datasets of the same sizes as in (*a*), except that in this case they are compiled over time points for fixed *n* since the TE is constant for times *t* > *n*.

We simulated a thousand trajectories lasting 50 time steps and then computed the mutual information for subsets of the data compiling 50, 450 and 950 agents for each time. Technically, data for different values of *n* and *t* are drawn from different distributions, but we can overcome this hurdle by taking advantage of several simplifying features of the model (see electronic supplementary material, S2 for details). The results are plotted in figure 10*a*, and it is clear that the smallest dataset (in red) is too noisy to robustly capture the temporal growth in the mutual information. Of equal note, however, is that the largest dataset (blue) only modestly improves the results obtained via the intermediate dataset (green), which already succeeds in capturing at least the qualitatively correct trend in the mutual information with time. For continuous random variables, especially, this may be the best we can expect to do. Estimating the underlying probability distribution functions of continuous random variables from data is most straightforwardly done through histogramming, and this discretization necessarily introduces inaccuracy into the IT calculation. To reproduce the formally correct result, one in principle needs to take the limit as the histogram bin size approaches zero, but populating such a histogram sufficiently would require an immense amount of data measured at a very high resolution. We used the Freedman–Diaconis rule in our numerical computation of the IT metrics in figure 10, as this method is easy to implement and has been shown to work well for unimodal distributions [52].

As another example, we also computed the transfer entropy ${\text{TE}}_{X_{n-1}\to X_{n}}(t)$ for different values of *n*. In this case, we simulated a chain of 100 ants for 1050 time steps and took advantage of the fact that, for *t* > *n*, the transfer entropy remains constant at its maximal value, thereby allowing us to compile data across time points *t* > 100 for each adjacent pair of ants. This theoretical limiting value of the transfer entropy, which is formally equal to (1/2) log (1 + *n*), is plotted in figure 10*b* for three datasets pooling across 50, 450 and 950 time points. The quality yielded by the three datasets follows the same trend as with the MI, which is surprising since transfer entropy usually requires estimating a joint distribution involving at least three random variables (compared to two for the MI). In fact, recall from §2.3 that the transfer entropy we computed formally depends upon four random variables: *X*_*n*_(*t*), *X*_*n*_(*t* − 1), *X*_*n*−1_(*t* − 1) and *X*_*n*−1_(*t* − 2). Datasets of the same size as those used to compute the MI ought to be insufficient for populating a four-dimensional histogram, but in this special case we have made use of the fact that ${\text{TE}}_{X_{n-1}\to X_{n}}$ is equal to MI(Δ*X*_*n*_(*t*); Δ*X*_*n*−1_(*t* − 1)), effectively reducing the number of random variables from four to two. While this is only possible because the underlying dynamics of the model are based on step sizes as opposed to absolute positions, this is not an uncommon feature of collective motion and may, therefore, be useful in other scenarios. In general, an organism’s absolute position will necessarily drift as it moves with its group, but quantities like its velocity or its distance to another organism, both of which depend upon a difference in positions, will often be more narrowly distributed and have a better chance of being stationary.

### 3.3. Caveats and concerns

As demonstrated in our last example, the IT tools we covered here, while potentially a powerful means to study information exchange in groups, are not universally appropriate and even Shannon urged caution among his peers [40]. IT tools can be deceptively difficult to apply and interpret, leading investigators to spurious conclusions [35–38]. Table 1 enumerates some of the most common challenges investigators encounter when attempting to use IT metrics and provides several possible solutions or workarounds for each. Each entry in the table is discussed and expanded upon in the paragraphs that follow.

**Table 1.** Common challenges and approaches.

challenges	approaches
data length and sampling interval	
	simulations
	*a priori* information
	movement scale
	optimization routines
discretization	binning optimization
	Kraskov method
equivalency	
	avoid data aggregation
stationarity	
	standard time-series approaches
	preference for relative over absolute quantities
	sub-setting or optimal partitioning
	time-varying metrics
reliability	
	comparative approach
	null models
	analytical expressions

#### 3.3.1. Data length

The simplest problem one can encounter with a dataset is that it is not a representative sampling of the relevant possibility space. This can be a problem in any statistical analysis, but it is especially severe in situations where two or more random variables need to be compared to one another, as is often the case in information theory. Even if one has adequately sampled the two random variables individually, their joint distribution, which is needed to compute comparative IT metrics like the mutual information, may still be sampled only sparsely. This problem is only exacerbated when one considers metrics like the transfer entropy or causation entropy, which require thorough samplings of joint distributions of three or more random variables. This increasing difficulty in sampling higher dimensional multivariate distributions is often called the ‘curse of dimensionality’ [86].

The most obvious way to lift this curse is to take more measurements from the system, by either performing a longer experiment, sampling more frequently or performing additional replicate experiments. The increased availability of various recording technologies would seem to address this data length problem by allowing investigators to log data at relatively high spatial and temporal frequencies for extended periods of time (ranging from seconds to days) [22,26,27,76,77,87,88]. Post-processing efforts to reduce errors from biological or mechanical processes, however, invariably reduce the amount of useable information and hours of data recording may result in tracks that, on average, last for less than a minute [89]. To date, most applications of IT metrics have been in laboratory studies where data length can be a more tractable problem [26,44], yet even here the amount of usable data points can be limited when the spatial or temporal event of interest is relatively small (e.g. maze studies [18,76]). In such cases, it may be possible to circumvent any data length issues by using *a priori* information to define the possibility space. For instance, in §3.2, we were able to use our knowledge of the system to both aggregate data across individuals and reduce the possibility space required for our IT metrics. While pooling data across individuals or events can be a common strategy for addressing one's data needs, this approach should be considered carefully (see §3.3.4).

#### 3.3.2. Sampling interval

The time interval between samples is closely linked to the issue of data length and plays an important role in determining both the amount and type of information available. This parameter will be defined by the rate at which the biological process of interest changes over time and it invariably impacts the values of the IT metrics. Sampling at too coarse an interval risks averaging over important dynamics, while sampling at too fine an interval can suggest more instances of a given state than are present. Both under- or over-sampling a time series can result in IT metrics identifying spurious links [37,90]. At the scale of moment-to-moment decisions, sampling intervals can be inferred from trends in the organisms’ movements that suggest how often individuals make course adjustments [26,60]. They have also been selected by sensitivity analyses [44] and optimization routines [27].

Other factors, like the scale of biological organization in question, the type of animal society being studied, or the ecological context in which the data are collected can also be expected to influence the various types of structural patterns that are revealed at different time scales. Animals with prior directional information in migrating populations, for instance, may have greater influence on the long-term movements of a group, but other individuals may display short-term influence during daily foraging expeditions [89,91]. Evidence of such age-structured effects on a social group’s movement dynamics is found across a wide range of animals [72].

#### 3.3.3. Discretization

The problems associated with data sampling are compounded by the fact that many, if not most, of the quantities of interest in the study of collective motion are continuous rather than discrete random variables. Positions, velocities and orientations, for example, are all continuous quantities whose likelihoods are formally determined by continuous probability distribution functions. Estimating one of these functions from a set of data is accomplished most straightforwardly by discretizing the random variable in question and treating its normalized histogram as a probability mass function. Some choices of discretization will yield more accurate approximations to the true distribution function, and numerous discretization schemes such as the empirically derived Freedman–Diaconis rule [52] and Scargle’s Bayesian blocks algorithm [92] have been developed to try to optimize this choice. Exploring how bin size impacts an IT metric of interest using simulations is also a sensible means of verifying bin size [44]. Another approach has been to select bin sizes based on the biometrics of the subject animals (e.g. setting bin widths to body lengths when discretizing speed data) [93].

The fundamental problem is that whereas simple statistics like the mean will be relatively insensitive to the details of the discretization scheme, so long as key features like peaks are adequately represented, the value of the Shannon entropy depends explicitly upon the number of discrete bins in the histogram. For continuous random variables, metrics like mutual information can be shown to only approach their formally correct values in the limit where the bin width approaches zero [34], a problem we demonstrated in the previous section with the numerical MI evaluation in our ant model. Shrinking the bin width to arbitrarily small sizes can lead to other problems, however. First, a histogram with more bins requires a larger volume of higher resolution data to adequately sample it. Second, the Shannon entropy actually diverges as the bin width shrinks to zero, and, although these divergences will always cancel out in the computation of quantities like mutual information, the subtraction of two very large numbers can result in computational precision errors.

One notable technique that avoids discretization entirely is the Shannon entropy estimator first devised by Kozachenko and Leonenko [94], which relies on a *k*-nearest neighbours algorithm instead of a histogram. Kraskov *et al*. [36] later generalized this method to estimate the mutual information. Far from a magic bullet, this method still relies upon a dense sampling of the possibility space, as one of its key approximations is that the probability distribution is constant over the volume containing each data point and its k-nearest neighbours. If the dataset is too sparse, this approximation obviously breaks down. The choice of *k* can also be more of an art than a science. Nonetheless, this method has been shown to provide a marked improvement in the results of transfer entropy calculations compared to methods that rely upon discretization schemes [95].

#### 3.3.4. Equivalency

When time-series data from *n* different processes (or animal tracks) are aggregated together in an attempt to better sample the dynamics, the assumption is implicitly being made that these processes are equivalent, i.e. each of the *n* processes is sampling the same underlying probability distribution. In some cases, investigators may be able to estimate the probabilities for each individual using only data from that individual’s track record [28,44], but keeping these tracks robustly differentiated becomes increasingly difficult as the size of the group and the complexity of the motion grows. When applying IT tools to larger groups, investigators may choose to restrict their efforts to a subset of the group [26] or aggregate the data across all individuals [27]. At the population scale, investigators may be forced to combine data [96] or rely on data that has already been aggregated [24]. Relying on individual trajectories to infer the underlying probabilities is preferable, while focusing on a subset of individuals raises the question as to how representative the sub-samples are of group-level structures.

For some insight into when it is reasonable to treat different individuals as equivalent, we look once more to our simple ant model. The distribution of the position of ant *n* at time *t*, *p*(*X*_*n*_(*t*)), can be shown to be a normal distribution with a variance that depends explicitly upon both *t* and *n*. If one were to try to deduce the form of this distribution from a time-series trajectory of the model, one might be tempted to aggregate the data for each time point and each ant together so as to maximize the sample size. But because the underlying distribution is not stationary, data from different time points actually sample from different distributions. The same holds for data points corresponding to different ants, because, formally, the ants are not statistically equivalent.

**Figure 11.**
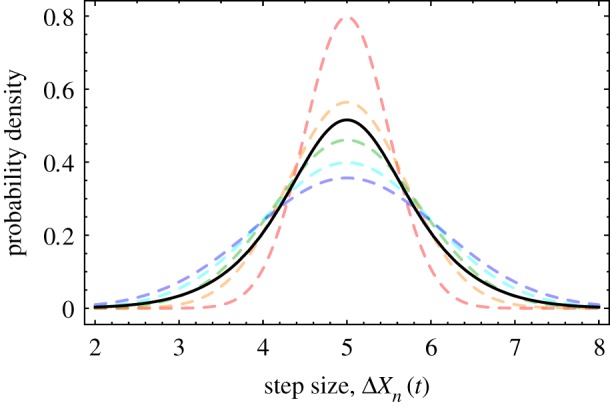
The effects of aggregating data across time points and agents. In the ant model, for a chain of five ants with a mean step size of five units, the step size Δ*X*_*n*_(*t*) can be shown to sample, for different values of *t* and *n*, from five different normal distributions, plotted as dashed curves. The black solid curve represents the mean of these distributions and is what one samples if the data for Δ*X*_*n*_(*t*) is aggregated across all values.

The result of naively aggregating data across time points and agents is illustrated in figure 11 for the ant model distribution *p*(Δ*X*_*n*_(*t*)). The data points from a trajectory of the system can be formally shown to sample from *N* different Gaussian distributions, where *N* is the total number of ants; the figure plots these distributions in different colours for *N* = 5. If all the data points of the trajectory are assumed to sample from the same distribution, the distribution that will be deduced from the analysis is actually the mean of the *N* underlying distributions, plotted in black in the figure. Pooling data across individuals should in principle only be done when this ‘mean’ distribution is expected to be a sufficiently good approximation to the distribution of each agent, but it is not apparent how severe a departure from this condition is necessary before one can expect deleterious effects on IT estimates.

#### 3.3.5. Stationarity

Stationarity suggests that the random variable(s) of interest that characterize the motion of an individual must be at a steady state. This assumption will impact IT estimates for both individual and aggregated data and there are a number of resources on identifying and dealing with issues of stationarity in times series [97,98]. In the aggregated case, such as with our Vicsek model example, the metric in question (orientation) tends to fluctuate randomly in time about a sharply peaked average, suggesting that its distribution is stationary. In some cases, we will not see such a clear signal, as a group’s behaviour will vary (i.e. drift) over time, and we must then attempt to find an optimal partitioning of the time series into datasets that are as long as possible without obscuring transitions between qualitatively distinct behavioural regimes [26,28,90,99]. We may also expect that persistent stationarity issues could be due to periodic patterns in an organism’s gait, in which case identifying and removing such trends may alleviate the problem.

Sometimes if a variable of interest is demonstrably not stationary, differencing can help. Consider that the distribution *p*(*X*_*n*_(*t*)) in our ant model is non-stationary, since the absolute position of each ant becomes increasingly uncertain in time; but the step size distribution *p*(Δ*X*_*n*_(*t*)) becomes stationary for *t* > *n* since the variance in an ant’s step size is ultimately just the sum of those of the ants in front of it. The value of such differencing may also result in a more biologically meaningful metric, as individuals appear to respond preferentially to the rate at which something is changing in their surroundings, rather than their current state (e.g. a preferential response to dynamic over static cues; fish [100] and humans [101]). Motion cues are an example of a dynamic visual feature that captures attention and such cues play an important role in influencing the neuromotor-actions of organisms (insects [102,103], fish [104–106], humans [107–109]).

A weaker condition for stationarity is essentially a ‘mean-field’ assumption that each agent should interact over time with the same average environment as every other agent. At any given time, each Vicsek agent, for example, will determine its next orientation by averaging over a different set of neighbouring orientations. If the system is sufficiently dense, however, the stochastic fluctuations of these random variables about the same mean will cancel each other out over time, making the mean-field assumption reasonable. It clearly does not hold for the ant model, on the other hand, since the hierarchical nature of the dynamics ensures that no two ants see an equivalent interaction environment. Similarly, when ants recruit others via a pheromone, the concentration of that chemical attractant will vary with traffic level, so that any mean-field assumption would fail over a long enough timeline.

#### 3.3.6. Reliability

In addition to the standard data preprocessing caveats reviewed above, there are also concerns related to the reliability of transfer and causation entropy values that should be considered when applying these metrics. For example, Smirnov demonstrated that if the temporal discretization of a dataset is too coarse to capture certain fundamental time scales of the sampled dynamics, one can end up computing non-zero transfer entropies between stochastic processes that are not causally linked [37]. Butail *et al.* [60] found support for these concerns in their attempts to use TE to quantify the extent to which fish-shaped robot confederates influenced the motion of actual fish; but, through repeated tests and parameter exploration, they were ultimately able to show a significant directional trend that matched their expectations. The drawbacks in using transfer entropy to identify persistent trends in leadership can also be reduced by combining TE with other statistical metrics, such as correlations and extreme event synchronization [23,44]. Missing covariates can also lead to spurious conclusions by suggesting associations between individuals whose apparent interactions arise only because they are each responding to another, unseen process. The gradient tracking examples in figure 7, for instance, illustrate how an underlying process can contribute to the group’s movement patterns. Runge [90] discusses the issue of missing (or latent) processes and discusses some potential solutions to identifying spurious links among entities.

Even the fundamental interpretation of transfer and causation entropy as measures of information flow has been called into question, based on the fact that conditioning one random variable on another is not formally equivalent to subtracting out its influence. James *et al.* [38] were able to contrive some simple examples where TE could not correctly account for the directed influence present, but they relied on situations where the uncertainty in a process is not reduced by knowing its past trajectory or where two processes can consistently influence a third process simultaneously but not individually. Fortunately, these particular conditions are unlikely to hold for organisms acting collectively, where knowledge of an individual’s past behaviours generally plays a prominent role in understanding current and future behaviours. Likewise, the qualitative dynamics of group cohesion are generally assumed to not depend upon irreducibly polyadic interactions, i.e. three or higher-body interactions that cannot be reduced to a sum of two-body interactions. This assumption has been validated in small to medium fish shoals [20], though it has not been ruled out that such interactions might be necessary to account for some more complex types of emergent social behaviour. Nonetheless, it is worth keeping in mind that IT metrics are better thought of as statistical scores than measurable observables. As such, we should proceed with due caution when applying and interpreting IT metrics, particularly when using them as primary response values in statistical analyses. The fickle nature of *p*-values [110] could just as easily be applied to IT metrics.

## 4. Conclusion

Identifying how the members of a group interact and coordinate their activity is a fundamental question of complex, self-organizing societies. Historically, some of the best examples for collective behaviour have come from the eusocial insects, where we find established connections between the individual sensory mechanisms and behavioural strategies that help shape the patterns we observe at the system level. Translating these lessons to other iconic examples of collective behaviours, like those observed in vast shoals of fish, murmurations of birds or crowds of pedestrians, has been far more challenging. Each ecological example of collective behaviour presents its own unique investigative challenges and any set of tools that may provide new insights merits attention. In this paper, we have discussed several tools based on Shannon’s information theory that are designed to quantify communication patterns, and we have demonstrated some of the benefits and pitfalls of applying these IT tools to group movement data.

Designing interaction rules based on mathematical and biological principles to replicate certain collective motions is a *forward problem* and a mature area of scholarship. Discerning interaction rules from data gathered on collective patterns, however, is a more recent and challenging class of *inverse problems*, and IT methods are one promising way to advance our understanding in this area. Advances in tracking technology [111], machine learning [112] and virtual reality [113,114] will further enable us to draw ties between individual- and group-level processes by streamlining data collection and advancing our experimental control over these dynamical systems. We must keep in mind, however, that while Shannon’s framework itself is over half a century old, its application to the study of collective phenomena is, by comparison, only in its infancy. The challenges we have outlined make it clear that reproducible insights will rely on a combination of these model-driven and model-free approaches. We have used simple examples to highlight some of these issues, but the solutions we have provided are only a starting point for further research efforts.

## Supplementary Material

Supplementary Material

## Data accessibility

This article does not contain any additional data.
