# Supplementary material for: Decoding collective communications using information theory tools
Source: J R Soc Interface. 2020 Mar 18;17(164):20190563. doi: 10.1098/rsif.2019.0563 (PMC7115225; doi:10.1098/rsif.2019.0563)
Supplement: Supplementary Material [file rsif20190563supp1.pdf]

Supplementary Information: Decoding collective communications using Information Theory tools

Pilkiewicz, K.R.*[1], Lemasson, B.H†[2], Rowland, M.A.[1], Hein, A.[3,4], Sun, J.[5], Berdahl, A.[6], Mayo, M.L.[1], Moehlis, J.[7], Porfiri, M.[8], Fernández-Juricic, E.[9], Garnier, S.[10], Bollt, E.M.[5], Carlson, J.M.[11], Tarampi, M.R.[12], Macuga, K.L[13], Rossi, L.[14], and Shen, C.-C.[15]

[1]*Environmental Laboratory, U.S. Army Engineer Research & Development Center (EL-ERDC), Vicksburg, MS, USA*
[2]*EL-ERDC, Newport, OR, USA*
[3]*National Oceanic and Atmospheric Administration, Santa Cruz, CA, USA*
[4]*University of California, Santa Cruz, Santa Cruz, CA, USA*
[5]*Department of Mathematics, Clarkson University, Potsdam, NY, USA*
[6]*School of Fisheries & Aquatic Sciences, University of Washington, Seattle, WA, USA*
[7]*Department of Mechanical Engineering, University of California, Santa Barbara, CA, USA*
[8]*Department of Mechanical and Aerospace Engineering and Department of Biomedical Engineering, New York University Tandon School of Engineering, Brooklyn, NY, USA*
[9]*Department of Biological Sciences, Purdue University, IN, USA*
[10]*Department of Biological Sciences, New Jersey Institute of Technology, Newark, NJ, USA*
[11]*Department of Physics, University of California, Santa Barbara, CA, USA*
[12]*Department of Psychology, University of Hartford, CT, USA*
[13]*School of Psychological Science, Oregon State University, OR, USA*
[14]*Department of Mathematical Sciences, University of Delaware, Newark, DE, USA*
[15]*Department of Computer and Information Sciences, University of Delaware, Newark, DE, USA*

January 15, 2020

*author for correspondence: Kevin.R.Pilkiewicz@usace.army.mil
†author for correspondence: brilraven@gmail.com

S1 Optimal Causation Entropy in a Nutshell

Suppose that we are given a set of time series $\{X_i(t)\}$ from a multivariate stochastic process ($i = 1,\dots,n$). Each $X_i(t)$ could represent, for example, the vector position of one bird within a flock at time t. Making the usual assumption of Markovity, we can assume that $X_j(t)$, for some specific j, is determined completely by the set $\{X_i(t-1)\}$. Due to the complex interdependence of these processes on one another, it is quite likely that much of the information flowing into $X_j(t)$ is redundant and that there is some reduced set $S_{t-1} \subset \{X_i(t-1)\}$ such that

$$P(X_j(t)|S_{t-1}) = P(X_j(t)|\{X_i(t-1)\}). \tag{1}$$

In general, there are likely many such sets that fulfill the above condition, but our goal is to identify the smallest such set. The members of that set are called the "causal parents" of $X_j(t)$. If the system described by $\{X_i(t)\}$ is in a stationary state, then X_j will have the same causal parents S at all times.

It can be shown that finding the set of causal parents S for each process $X_j(t)$ is equivalent to finding the smallest subset S'_{t-1} of $\{X_i(t-1)\}$ that maximizes the so-called "unconditional" causation entropy

$$C_{S'\to j} \equiv MI(X_j(t);\, S'_{t-1}). \tag{2}$$

Note that this unconditional causation entropy differs from the standard causation entropy defined in Eq. (14) of the main text. Finding the smallest set S' that maximizes this information theory metric is a nonconvex optimization problem, but a simple, two-stage algorithm, dubbed the optimal causation entropy (oCSE) algorithm, has been shown to suffice.

The first, forward stage of the oCSE algorithm iteratively finds, one at a time, the process $X_i(t-1)$ that maximizes the incremental reduction of uncertainty with regards to the target variable $X_j(t)$, until no further reduction is possible. The outcome of the first stage is a set of candidate causal parents that includes the true causal parents but may also contain other members of $\{X_i(t-1)\}$ that are "false positives". In the second, backward stage, the algorithm examines each member of the candidate set to remove those that are redundant. When the exact distributions and entropies are known, the resulting set returned by the algorithm is the true set of causal parents. In practical applications where these distributions and entropies must also be estimated from the data, the outcome of the oCSE algorithm only provides an estimate of the true causal parents.

S2 Aggregating data: an example from the ant model

Aggregating group data across different time points or agents can be a useful trick for achieving a large sample size from a limited amount of experimental data, but oftentimes this cannot be done because the distributions underlying the random variables of interest are nonstationary or differ from agent to agent. In the ant model, for example, most of the distributions generally depend upon both the time t and the ant index n, thereby severely reducing the amount of available data for computing any IT metrics of interest. For the mutual information $MI(X_n(t);X_n(t-1))$ computed in section 2.2 of the main text (see figure 3 B), we can get around this issue by noting several important properties of the model. First, for $t \leq n$, the MI is equal to $(1/2)\log t$ and thus depends only on time. Second, because the random variables in this model are all normally distributed, the entropies needed to compute the MI will depend only on the variances of the corresponding probability distributions and not their means [1]. As a result of these properties, we can shift the values of the ant positions so that $X_n(t)$ is distributed about zero mean for all n and t and then, for each time point, pool together the shifted positions of each ant for which $n > t$. In most real systems, this degree of "inside" knowledge would not be available, and the potential benefits of aggregating data to improve sampling would have to be weighed carefully against the potential costs of wrongly comparing apples to oranges.

References

[1] Cover T, Thomas J. 2006 *Elements of information theory*. Wiley-Interscience, Hoboken, NJ 2nd edition.
